# Integrated Bioinformatics Analysis Reveals the Impact of SHEV ORF3-Related LncRNA Network on Bile Secretion Pathway (ko 04976) in HepG2 Cells

**DOI:** 10.3390/vetsci13030276

**Published:** 2026-03-16

**Authors:** Hanwei Jiao, Jiya Li, Shengping Wu, Lingjie Wang, Yu Zhao, Yulong Yin, Xin Cao, Leli Wang

**Affiliations:** 1 The College of Veterinary Medicine, Southwest University, Chongqing 402460, China; lijiya1125@outlook.com (J.L.); chemie@email.swu.edu.cn (S.W.); guolicheng666@email.swu.edu.cn (L.W.); 2 College of Veterinary Medicine, Jilin Agricultural University, Changchun 130118, China; xinc@jlau.edu.cn; 3 Institute of Animal Husbandry and Veterinary Medicine of Guizhou Academy of Agricultural Science, Ministry of Agriculture and Rural Affairs Key Laboratory of Crop Genitic Resources and Germplasm Innovation in Karst Region, Guiyang 550005, China; zhaoyu@gzsnky.wecom.work; 4 Institute of Subtropical Agriculture, Chinese Academy of Sciences, Changsha 410125, China; yinyulong@isa.ac.cn

**Keywords:** SHE, SHEV ORF3, HepG2, bile secretion, lncRNA-mRNA network

## Abstract

(1) Background: Swine hepatitis E (SHE) is an emerging zoonotic disease caused by the swine hepatitis E virus (SHEV). The open reading frame 3 (ORF3) protein is a recognized virulence factor of SHEV. Jaundice, the typical clinical sign of SHE, primarily results from disruptions in bile production, secretion, and excretion. However, the mechanism by which SHEV ORF3 influences bile metabolism remains unclear. (2) Methods: Building on our previous work involving adenovirus-mediated overexpression of genotype IV SHEV ORF3 in HepG2 cells and subsequent high-throughput lncRNA/transcriptome sequencing, this study performed KEGG enrichment analysis on differentially expressed lncRNAs. Candidate lncRNAs were validated via qRT-PCR. Cis-regulated target genes were predicted by integrating differentially expressed mRNA data. Furthermore, AlphaFold 3.0 was employed to analyze the molecular binding sites between lncRNA UBC (MSTRG.6881.4) and its target, UBC protein. (3) Results: We identified three lncRNAs associated with the bile secretion pathway (ko 04976) in HepG2 cells expressing genotype IV SHEV ORF3, which were further confirmed by qRT-PCR: lncRNA UBC (MSTRG.6881.4), lncRNA UBC (MSTRG.6881.9), and lncRNA UBC (MSTRG.6881.12). Bioinformatics prediction suggested six lncRNA-mRNA regulatory networks involved these lncRNAs and two downregulated UBC mRNA transcripts (ENST00000540700 and ENST00000536769). Molecular docking indicated that nucleotides 395U and 41C of lncRNA UBC (MSTRG.6881.4) could potentially bind to residues 82Lys, 88Thr, and 90Thr of the UBC protein, with predicted binding energies ranging from −4.73 to −0.75 kcal/mol. (4) Conclusions: The successful identification of bile secretion-related lncRNAs, coupled with the prediction of their regulatory networks and molecular interaction sites, has advanced our understanding of SHEV ORF3 function and the pathogenesis of SHEV infection.

## Simple Summary

Swine hepatitis E (SHE), caused by the swine hepatitis E virus (SHEV), is a significant zoonotic disease which is often associated with disrupted bile secretion. This study focused on the role of the SHEV ORF3 protein in this pathological process. Using bioinformatics approaches on HepG2 cells expressing genotype IV SHEV ORF3, we identified three specific long non-coding RNAs (lncRNAs) linked to the bile secretion pathway. We further constructed their potential regulatory networks with target mRNAs and characterized putative molecular binding sites. These findings could offer novel insights into the functions of the ORF3 protein and the infection mechanism of SHEV.

## 1. Introduction

The hepatitis E virus (HEV) is the most common pathogen causing acute viral hepatitis worldwide. Hepatitis E (HE) is an important public health problem in developing countries, causing a serious economic burden [1,2,3]. Swine are natural hosts of HEV, and their infection rate is much higher than other animals. Mainly infecting swine aged 2–6 months, it can be detected in the small intestine, liver, colon, etc. But HEV has weak resistance to the outside world, and HEV in pork products can be killed by frying or boiling for 5 min. As a zoonotic disease, swine infected with the swine hepatitis E virus (SHEV) are the main hosts of human HEV contamination after fecal–oral transmission [4,5,6].

The HEV genome, approximately 7.2 kb in length, comprises three open reading frames (ORF1, ORF2, and ORF3). ORF1 encodes non-structural proteins, including enzymes involved in viral replication and transcription. ORF2 encodes the viral capsid protein, while ORF3 encodes a small phosphoprotein. Although the ORF2 protein, as a key viral antigen and structural component, has been extensively characterized, research on the ORF3 protein has intensified in recent years. ORF3 is recognized as a multifunctional protein essential for viral infectivity and egress, and it participates in modulating host cell signaling pathways [7,8,9].

A defining clinical manifestation of swine hepatitis E (SHE) is jaundice accompanied by hepatitis-related pathological alterations in the liver. Jaundice, also known as cholestasis, can be caused by two main reasons: disorders in the production, secretion, and excretion of bile, and disorders in the production and metabolism of bilirubin. The formation process of jaundice is very complex, and its molecular regulatory mechanism is not fully understood [10,11]. Studies are being conducted into how the HEV (especially genotype 3/4) interacts with the complex material transport network (uptake, intracellular transport, excretion) of liver cells during infection to complete its lifecycle; at the same time, research is also being conducted on how therapeutic drugs reach their targets through this network, as well as the possible “transport competition” relationship between viruses and drugs.

Long non-coding RNAs (lncRNAs), transcripts longer than 200 nucleotides with limited protein-coding potential, play crucial roles in diverse physiological and pathological processes, including disease progression, autophagy, apoptosis, and cell differentiation. Consequently, they have become a focal point in transcriptional regulation research [12,13,14]. This study leverages transcriptomic data from HepG2 cells with adenovirus-mediated overexpression of genotype IV SHEV ORF3. Through Gene Ontology (GO) and Kyoto Encyclopedia of Genes and Genomes (KEGG) enrichment analyses, we have identified, for the first time, lncRNAs exhibiting significant differential expressions within the bile secretion pathway and predicted their target genes. Given that bile secretion dysfunction is a direct cause of jaundice—the primary clinical symptom of SHEV infection—this work aims to delineate the lncRNA-mRNA regulatory networks within the bile secretion pathway modulated by SHEV ORF3 in HepG2 cells. Our findings can establish a foundation for future investigations into the functional role of SHEV ORF3 and the infectious mechanism of SHEV.

## 2. Materials and Methods

### 2.1. ORF3 Overexpression and RNA Sequencing

HepG2 cells (Shanghai Cell Bank, Chinese Academy of Sciences) were infected with recombinant adenoviruses AD-ORF3 or AD-GFP (constructed by Shanghai GenePharma Co., Ltd., Shanghai, China, as referenced in our previous study) at a multiplicity of infection (MOI) of 10. The overexpression of SHEV ORF3 protein was confirmed by Western blot analysis as described in our previous study. Total RNA was extracted 48 h post-infectionusing TRIzol™ Reagent (Invitrogen, Thermo Fisher Scientific, Waltham, MA, USA) for high-throughput sequencing of lncRNA and transcriptome [15,16].

### 2.2. Bioinformatics Analysis

**Figure 1:**
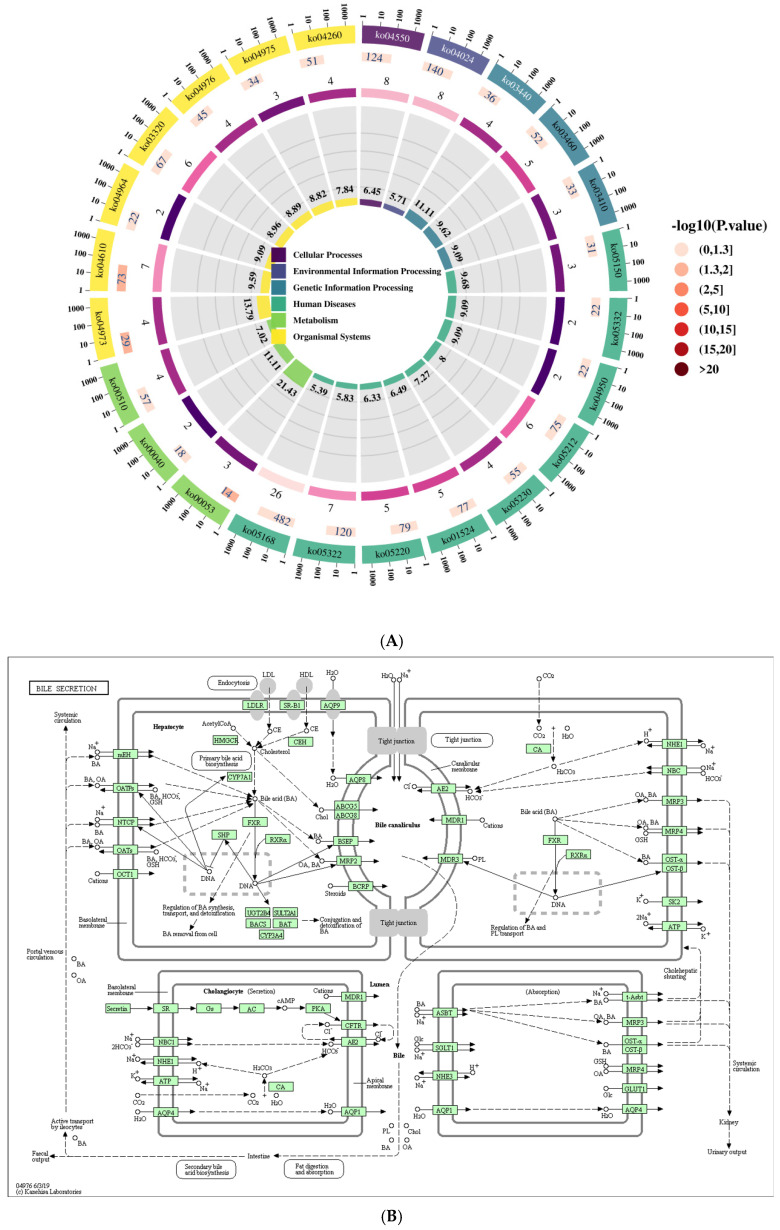
Identification of the bile secretion pathway (ko 04976) in genotype IV swine hepatitis E virus ORF3-expressing HepG2 cells. (**A**) KEGG functional cycle enrichment plot displays the top 24 pathways (*p* ≤ 0.05) and reveals the bile secretion pathway (ko 04976). (**B**) Diagram of the bile secretion pathway (ko 04976).

Sequencing reads were assembled using the StringTie software (v2.2.1). Known mRNA transcripts and those shorter than 200 bp were subsequently filtered out. Transcripts predicted to have coding potential by the Coding Potential Calculator 2 (CPC2) and Pfam database scans were also removed. The remaining transcripts were classified as lncRNAs. Following a series of filtering steps, a final set of significantly differentially expressed lncRNAs was obtained [15]. KEGG pathway enrichment analysis was performed on these lncRNAs, with the top 24 significantly enriched pathways (*p* ≤ 0.05) displayed. The bile secretion pathway diagram was analyzed in the context of other differentially enriched pathways. In these diagrams (Figure 1A), red denotes significantly differentially expressed genes annotated to a specific KEGG orthology (ko) node that are upregulated; blue indicates downregulated genes; and yellow represents genes within the same node showing both up- and downregulation. In addition, boxes contain Enzyme Commission (EC) numbers, hollow circles signify small molecules, solid arrows depict biochemical reaction directions, and dashed arrows link to associated metabolic pathways (Figure 1B).

**Figure 2:**
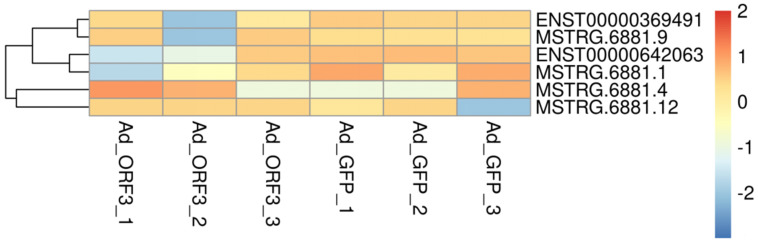
Identification of bile secretion pathway-associated lncRNAs from transcriptomic sequencing data in ORF3-expressing HepG2 cells.

Cluster analysis was performed on significantly differentially expressed lncRNAs within the bile secretion pathway, based on gene expression profiles from six samples (Ad_GFP1, Ad_GFP2, Ad_GFP3, Ad_ORF3_1, Ad_ORF3_2, Ad_ORF3_3). Their expression patterns across samples were visualized using a heatmap. The horizontal axis represents samples, and the vertical axis represents lncRNAs and mRNAs. A color gradient indicates expression levels, with red representing high expression and dark blue representing low expression (Figure 2).

### 2.3. qRT-PCR Validation

Design primers were based on the sequence of six lncRNAs of the bile secretion (ko 04976) pathway and qRT-PCR was performed to validate the six lncRNAs; the validation method was described before [16]. Briefly, cDNA was synthesized using the PrimeScript RT Reagent Kit (Takara Bio, Kusatsu, Japan). qRT-PCR was then performed using TB Green Premix Ex Taq II (Takara Bio) on a QuantStudio 3 Real-Time PCR System (Applied Biosystems, Waltham, MA, USA), with UBC (Ubiquitin C) used as the internal reference gene.

The primer sequences used in this study are listed in Table 1.

### 2.4. Cis-Regulatory lncRNA-mRNA Network Analysis in Bile Secretion

This study focused on cis-regulatory analysis of lncRNAs, which involved the regulation of neighboring genes. The prediction of cis-target genes was based on the differential expressions of lncRNAs and mRNAs within a 100 kbp genomic window upstream and downstream. As reported previously [16], transcriptome sequencing identified 217 differentially expressed genes (1379 transcripts) in HepG2 cells expressing genotype IV SHEV ORF3. Therefore, we integrated the analysis of lncRNAs within the bile secretion pathway with these transcriptome sequencing results to predict cis-target genes for the lncRNAs and to explore potential lncRNA-mRNA regulatory networks.

### 2.5. LncRNA-mRNA Molecular Docking Analysis

RNA-RNA interaction prediction was performed using IntaRNA 2.0 via the Freiburg RNA Tools webserver (https://rna.informatik.uni-freiburg.de/ (accessed on 22 January 2026)). IntaRNA 2.0 was run with default parameters, evaluating interaction stability while considering the accessibility of interacting subsequences. Following validation, IntaRNA 2.0 has proven to be a rapid and accurate tool for such predictions. The binding affinities (in kcal/mol) reported for RNA-RNA complexes were derived directly from the IntaRNA 2.0 predictions. For RNA–protein interaction analysis, AlphaFold 3.0 (https://alphafoldserver.com (accessed on 20 January 2026)) was employed primarily to predict the three-dimensional binding conformation between the selected lncRNAs and their target proteins. The resulting complex structures were visualized using PyMOL (v3.1.4.1). The predicted binding affinities (in kcal/mol) for the RNA–protein complexes were obtained from the interface scores provided by AlphaFold 3.0. The binding affinities for RNA–protein complexes were reported in kcal/mol, as provided by the interface scoring function of AlphaFold 3.0.

## 3. Results

### 3.1. Identification of the Bile Secretion Pathway Through KEGG Analysis

KEGG functional enrichment analysis revealed the top 24 significantly enriched pathways (*p* ≤ 0.05), including systemic lupus erythematosus (ko 05322); *Staphylococcus aureus* infection (ko 05150); signaling pathways regulating stem cell pluripotency (ko 04550); PPAR signaling pathway (ko 03320); platinum drug resistance (ko 01524), pentose and glucuronate interconversions (ko 00040); pancreatic cancer (ko 05212); N-glycan biosynthesis (ko 00510); homologous recombination (ko 03440); herpes simplex virus 1 infection (ko 05168); fat digestion and absorption (ko 04975); Fanconi anemia pathway (ko 03460); complement and coagulation cascades (ko 04610); central carbon metabolism in cancer (ko 05230); cardiac muscle contraction (ko 04260); carbohydrate digestion and absorption (ko 04973); cAMP signaling pathway (ko 04024); bile secretion (ko 04976); base excision repair (ko 03410); ascorbate and aldarate metabolism (ko 00053); chronic myeloid leukemia (ko 05220); proximal tubule bicarbonate reclamation (ko 04964); graft-versus-host disease (ko 05332); and maturity-onset diabetes of the young (ko 04950) (Figure 1A and Table 2). Consequently, KEGG analysis of potential target genes for differentially expressed lncRNAs identified the bile secretion pathway (ko 04976) as one significantly associated with lncRNA activity in HepG2 cells expressing genotype IV SHEV ORF3.

During the formation of bile acid, as shown by the bile secretion pathway (ko 04976) diagram in Figure 1B, the expression of mEH, OATPs, NTCP, OATs, OCT1, and CYP7A1 were significantly reduced in genotype IV SHEV ORF3-expressing HepG2 cells. The significant decrease in FXR gene expression can affect the regulation of bile acid synthesis, transport, and detoxification, as well as the clearance of bile from genotype IV SHEV ORF3-expressing HepG2 cells. When bile acids moved towards the bile duct and secrete bile, the expression levels of BSEP and MRP2 genes were significantly downregulated.

### 3.2. Screening of lncRNAs with Significant Differential Expression in the Bile Secretion Pathways (ko 04976)

In HepG2 cells, we revealed 62 significantly differentially expressed mRNAs (6564 transcripts) and 319 lncRNAs (124 known lncRNAs and 195 novel lncRNAs) which were mediated by genotype IV SHEV ORF3, as described before [15]. KEGG functional enrichment analysis performed on the potential cis-target genes of these differentially expressed lncRNAs identified six lncRNAs associated with the bile secretion pathway (ko04976): lncRNA ATP1A1-AS1 (ENST00000369491); lncRNA UBC (MSTRG.6881.9); lncRNA AL139011 (ENST00000642063); lncRNA UBC (MSTRG.6881.1); lncRNA UBC (MSTRG.6881.4); and lncRNA UBC (MSTRG.6881.12) (Figure 2 and Table 3).

The genomic coordinates (GRCh38/hg38) for these six candidate lncRNAs are as follows: ATP1A1-AS1 (ENST00000369491): chr1:116405509–116418536 (−); UBC (MSTRG.6881.1): chr12:124911832–124913709 (+); UBC (MSTRG.6881.4): chr12:124912060–124913707 (+); UBC (MSTRG.6881.9): chr12:124911832–124913707 (+); UBC (MSTRG.6881.12): chr12:124911832–124913709 (+); and AL139011 (ENST00000642063): chr1:160262015–160262720 (+).

### 3.3. qRT-PCR Validation of Six lncRNAs in the Bile Secretion Pathway (ko 04976)

**Figure 3:**
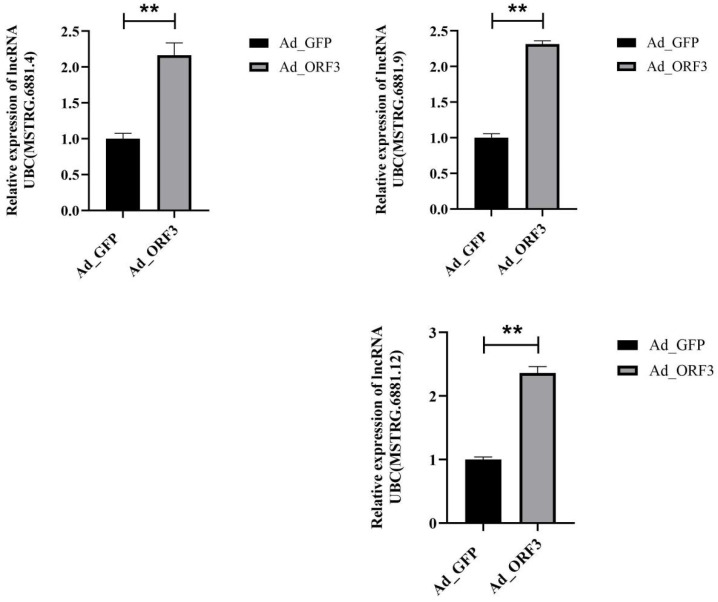
The six lncRNAs of the bile secretion (ko 04976) pathway were validated by qRT-PCR and three of them were consistent with the lncRNA transcriptomic results. ** *p* < 0.01 versus the Ad_GFP control group.

We performed qRT-PCR to validate the six lncRNAs in bile secretion pathway (ko 04976), FPKM (fragments per kilobase of exon model per million mapped reads) value can be equivalent to the expression level of genes in different samples. Log2 (fold change) was the FPKM value of AD_ORF3 divided by the FPKM value of AD_GFP. The validation results indicated that three lncRNAs were consistent with the lncRNA sequencing data, they were lncRNA UBC (MSTRG.6881.4), lncRNA UBC (MSTRG.6881.9) and lncRNA UBC (MSTRG.6881.12) (Figure 3).

### 3.4. Prediction of lncRNA-mRNA Networks

**Figure 4:**
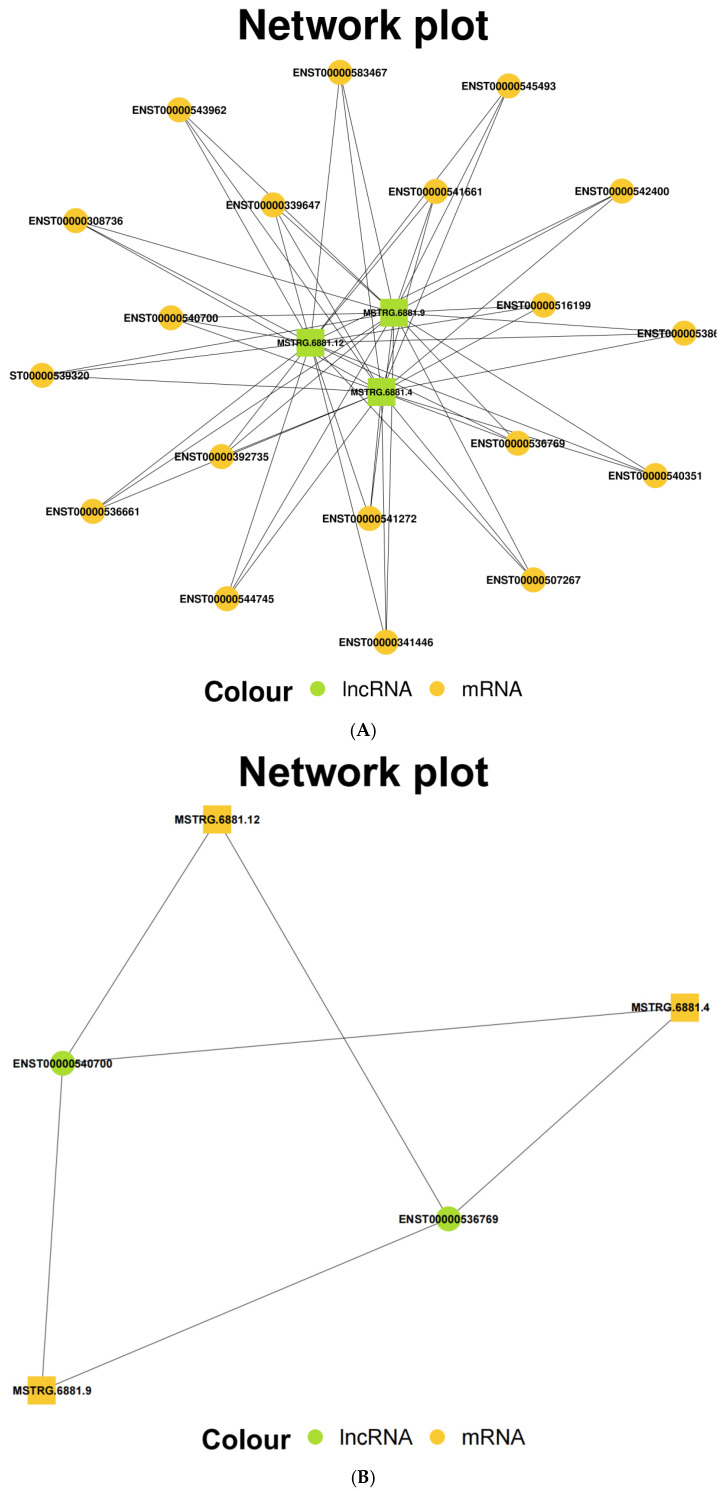
The three validated lncRNAs of the bile secretion (ko 04976) pathway were predicted from their target mRNA from transcriptome sequencing. (**A**) The three validated lncRNAs were predicted from the lncRNA-mRNA networks and their target mRNAs from transcriptome sequencing. (**B**) The three validated lncRNAs were predicted from the lncRNA-mRNA networks and their significantly differentially expressed target mRNAs from transcriptome sequencing.

Target gene prediction was conducted for the three validated lncRNAs: lncRNA UBC (MSTRG.6881.4), lncRNA UBC (MSTRG.6881.9), and lncRNA UBC (MSTRG.6881.12). As noted earlier [16], transcriptome sequencing identified 217 differentially expressed genes (1379 transcripts) in the experimental cells. Prediction analysis indicated 19 mRNAs as potential targets of these three lncRNAs (Figure 4A), with two mRNAs—ENST00000540700 and ENST00000536769—exhibiting significant differential expression (Table 4). Consequently, six potential lncRNA-mRNA regulatory networks were predicted: lncRNA UBC (MSTRG.6881.4)-ENST00000540700; lncRNA UBC (MSTRG.6881.4)-ENST00000536769; lncRNA UBC (MSTRG.6881.9)-ENST00000540700; lncRNA UBC (MSTRG.6881.9)-ENST00000536769; lncRNA UBC (MSTRG.6881.12)-ENST00000540700; and lncRNA UBC (MSTRG.6881.12)-ENST00000536769 (Figure 4B).

### 3.5. Molecular Docking Analysis

**Figure 5:**
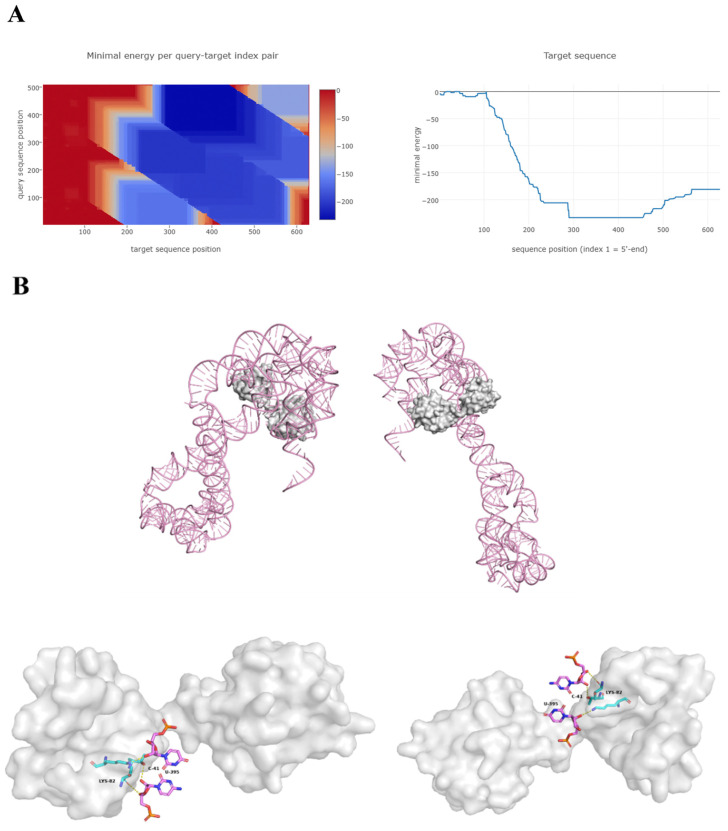
Prediction and molecular modeling of lncRNA UBC (MSTRG.6881.4)-UBC complex. (**A**) RNA-RNA binding site prediction of lncRNA UBC (MSTRG.6881.4)-UBC mRNA based on IntaRNA2.0. (**B**) RNA–protein binding site prediction and molecular modeling of lncRNA UBC (MSTRG.6881.4)-UBC protein based on AlphaFold 3.0.

Molecular docking analysis was performed on the highest expressed lncRNA, UBC (MSTRG.6881.4), and its potential target gene, UBC mRNA (ENST00000540700). Based on their nucleotide sequences, the Freiburg RNA Tools software predicted two RNA-RNA binding sites, nucleotides 345–493 and 171–320 of lncRNA UBC (MSTRG.6881.4), potentially interacting with nucleotides 305–453, 599–639, and 478–627 of UBC mRNA. The minimum binding energy for these interactions ranged from −233.35 to −180.83 cal/mol (Table 5, Figure 5A). The lncRNA UBC (MSTRG.6881.4)-UBC mRNA complex was modeled, and a binding energy heatmap illustrated the lowest energy distribution.

For RNA–protein interaction, AlphaFold 3.0 predicted binding between lncRNA UBC (MSTRG.6881.4) and the UBC protein. Modeling suggested three binding sites, with nucleotides 395U and 41C of the lncRNA potentially targeting residues 82Lys, 88Thr, and 90Thr of the UBC protein. The predicted minimum binding energy for these interactions ranged from −4.73 to −0.75 kcal/mol (Table 6, Figure 5B). Interaction types were classified as hydrophobic (D) and hydrogen bonding (H). The comprehensive prediction results propose specific molecular binding sites through which lncRNA UBC (MSTRG.6881.4) may target and regulate UBC.

### 3.6. Potential Impact of ORF3 Expression on the m6A Modification Landscape

To investigate whether the dysregulation of the identified bile secretion-associated lncRNAs involved upstream epigenetic regulation, we analyzed parallel transcriptomic data from the same ORF3-overexpressing cellular model [17]. The data revealed a downregulation trend in the expression of the m6A demethylase FTO [18,19]. Given that FTO is a key “eraser” of m6A, its functional suppression can lead to aberrant accumulation of m6A modification on RNA molecules [18,19]. The significant downregulation of key bile secretion pathway gene transcripts, such as UBC as observed in this study, may be partly attributed to increased m6A modification levels mediated by FTO inhibition, which confers transcript instability. This can provide a potential epigenetic explanatory dimension for how ORF3 disrupts bile acid metabolism.

## 4. Discussion

Swine hepatitis E (SHE), first identified by Meng et al. [20] and caused by the swine hepatitis E virus (SHEV), has shown a trend toward global dissemination, with cases reported in an increasing number of developed countries and regions. Consequently, SHE has emerged as a public health concern worldwide [21,22,23]. SHEV is categorized into four genotypes, with genotype IV first detected in the Chinese population in 1993 and predominant in Chinese swine farms—often termed the “Chinese genotype” [24]. The ORF3 gene, approximately 365 bp in length and partially overlapping with ORF2, encodes a phosphoprotein implicated in regulating viral-specific immune responses and cytoskeleton organization [25].

The formation of hepatocellular cholestasis may be caused by defects in the bile production function of hepatocytes, or by dysfunction in bile duct secretion. After the formation of hepatocytes, bile can be regulated and modified by the absorptive and secretory transport system of the bile duct epithelial cell cavity facial mask, which is then transported by the ion pump and dynamic molecular system to complete the secretion process of bile. This process is quite complex, and its molecular regulation mechanism is still unclear [26,27,28]. Infection with swine hepatitis E virus (SHEV) can lead to jaundice in pigs, primarily through virus-induced liver damage that disrupts bilirubin metabolism. Swine are natural hosts of HEV as they have a higher infection rate, especially during the piglet stage. HEV can mainly infect the small intestine, liver, and colon of swine. After infection, swine may experience jaundice symptoms, manifested as yellowing of the skin and conjunctiva [29,30].

Long non-coding RNAs (lncRNAs) are crucial regulators of diverse physiological and pathological processes [31]; however, their role in SHEV infection remains largely unexplored. This study was the first to identify key lncRNAs—UBC (MSTRG.6881.4, MSTRG.6881.9, and MSTRG.6881.12)—whose potential target genes were associated with the bile secretion pathway (ko 04976) in the context of genotype IV SHEV ORF3 expression. Ubiquitin C (UBC), a core component of the ubiquitin–proteasome system, is a small protein that tags target proteins for degradation via a cascade of enzymatic reactions, a process known as ubiquitination [32,33,34]. To our knowledge, no prior studies have reported a connection between UBC and SHE. Thus, our discovery can offer a novel perspective for investigating the mechanisms of SHE infection and the function of the SHEV ORF3 protein.

It is important to note that KEGG pathway enrichment analysis is based on the coding genes (mRNAs) associated with the differentially expressed lncRNAs, not the lncRNAs themselves. The identification of UBC, a core component of the ubiquitination system, within the context of the bile secretion pathway suggests a potential crosstalk between ubiquitin-mediated protein degradation/signaling and bile acid metabolism/transport. Although the dramatic downregulation of specific UBC transcripts (e.g., ENST00000540700 with log2FC of −15.97) was consistently observed across biological replicates in our sequencing data, its biological significance and whether it reflects a genuine phenomenon or potential technical artifact require further experimental validation, such as independent qRT-PCR for these specific UBC transcripts and functional assays.

It should be noted that the findings of this study were primarily based on bioinformatics prediction and molecular docking analysis. The predicted lncRNA-mRNA regulatory networks and binding sites require further experimental validation through functional gain- or loss-of-assays (e.g., overexpression, knockdown, dual-luciferase reporter assays) in cellular or animal models.

This study revealed the association between ORF3 and bile secretion-related lncRNA UBC networks. In light of the parallel research trend showing that ORF3 expression correlates with the downregulation of FTO [17], we proposed an integrated two-tier “epigenetic–transcriptional” regulatory hypothesis. At the epigenetic level, ORF3 may inhibit FTO, leading to global or transcriptome-specific m6A hypermethylation, thereby creating a background of “metabolic gene mRNA instability.” At the transcriptional/post-transcriptional level, specific lncRNA networks, such as UBC, can be activated or suppressed in this context [18], further fine-tuning the expression of downstream genes involved in bile acid synthesis and transport (e.g., BSEP, MRP2) through mechanisms like cis-regulation [35]. ORF3 may therefore disrupt bile acid homeostasis, ultimately leading to bile secretory dysfunction, via the synergistic dual mechanisms of “m6A-mediated global disturbance” and “lncRNA-mediated targeted regulation.” Future studies could employ techniques such as MeRIP-qPCR to directly validate changes in m6A modification on key gene transcripts [35], thereby clarifying the specific details of this regulatory pathway.

## 5. Conclusions

Our KEGG analysis initially identified six lncRNAs potentially linked to the bile secretion pathway (ko 04976) in HepG2 cells expressing genotype IV SHEV ORF3. qRT-PCR validation confirmed the differential expression of three novel lncRNAs: UBC (MSTRG.6881.4), UBC (MSTRG.6881.9), and UBC (MSTRG.6881.12). We predicted six regulatory networks between these lncRNAs and two significantly downregulated UBC mRNA transcripts. Molecular docking analysis suggested that nucleotides 395U and 41C of lncRNA UBC (MSTRG.6881.4) may bind to residues 82Lys, 88Thr, and 90Thr of the UBC protein. These results have provided a foundation for elucidating the function of SHEV ORF3 and the infection mechanism of SHEV.

## Figures and Tables

**Table 1:** Primer sequences for qRT-PCR validation.

lncRNA Gene ID	lncRNA Gene Name	Primers	Primer Sequences
ENST00000369491	*ATP1A1-AS1*	Forward primer Reverse primer	5′-TGTGAGGGCCGAGTGAAATC-3′ 5′-GAAGACCAACGCACATACGC-3′
ENST00000642063	*AL139011*	Forward primer Reverse primer	5′-CCAACGGAAAACAGAGCGAG-3′ 5′-CGGCAAAACTGGCCATCATC-3′
MSTRG.6881.9	*UBC*	Forward primer Reverse primer	5′-TCGGCTCCACTTCGAGA-3′ 5′-ATGGGCGCACCCTGTC-3′
MSTRG.6881.1	*UBC*	Forward primer Reverse primer	5′-GATGCCTTCCTTGTCTTG-3′ 5′-ATGGTCGTACCCTGTCTG-3′
MSTRG.6881.4	*UBC*	Forward primer Reverse primer	5′-GCAGGGTGGACTCTTTCT-3′ 5′-AGAGGCTGATCTTTGCTG-3′
MSTRG.6881.12	*UBC*	Forward primer Reverse primer	5′-CCCACCTCTAAGACGGAGCA-3′ 5′-CTGGAAGATGGACGCACC-3′

**Table 2:** Top 24 pathways identified by KEGG functional enrichment analysis (*p* ≤ 0.05), including the bile secretion pathway (ko 04976).

Pathway_ID	Pathway_Name
ko05322	Systemic lupus erythematosus
ko05150	*Staphylococcus aureus* infection
ko04550	Signaling pathways regulating pluripotency of stem cells
ko03320	PPAR signaling pathway
ko01524	Platinum drug resistance
ko00040	Pentose and glucuronate interconversions
ko05212	Pancreatic cancer
ko00510	N-glycan biosynthesis
ko03440	Homologous recombination
ko05168	Herpes simplex virus 1 infection
ko04975	Fat digestion and absorption
ko03460	Fanconi anemia pathway
ko04610	Complement and coagulation cascades
ko05230	Central carbon metabolism in cancer
ko04260	Cardiac muscle contraction
ko04973	Carbohydrate digestion and absorption
ko04024	cAMP signaling pathway
ko04976	Bile secretion
ko03410	Base excision repair
ko00053	Ascorbate and aldarate metabolism
ko05220	Chronic myeloid leukemia
ko04964	Proximal tubule bicarbonate reclamation
ko05332	Graft-versus-host disease
ko04950	Maturity-onset diabetes of the young

**Table 3:** Sequencing data of the six lncRNAs of the bile secretion pathway (ko 04976).

lncRNA Gene ID	lncRNA Gene Name	Known/Novel	Cis/Trans	Log2 (Fold Change)
ENST00000369491	ATP1A1-AS1	Known	cis	−1.20
MSTRG.6881.9	UBC	Novel	cis	1.27
ENST00000642063	AL139011	Known	cis	−1.88
MSTRG.6881.1	UBC	Novel	cis	−1.64
MSTRG.6881.4	UBC	Novel	cis	3.62
MSTRG.6881.12	UBC	Novel	cis	1.70

**Table 4:** Sequencing data of two target mRNAs of the validated three lncRNAs of the bile secretion pathway (ko 04976).

mRNA Transcript	Description	Regulation	Significant	Log2 (Fold Change)
ENST00000540700	ubiquitin C	down	yes	−15.97
ENST00000536769	ubiquitin C	down	yes	−6.18

**Table 5:** IntaRNA2.0 prediction of lncRNA UBC (MSTRG.6881.4)-UBC mRNA RNA-RNA binding site.

Transcript	Start (Q)	End (Q)	Gene	Start (Q)	End (Q)	Energy (cal/mol)
MSTRG.6881.4	345	493	ENST00000540700	305	453	−233.35
	171	320		478	627	−180.83

**Table 6:** AlphaFold 3.0 prediction of lncRNA UBC (MSTRG.6881.4)-UBC RNA–protein binding site.

Transcript	Position	Set (dNTP)	Protein	Position	Set (AA)	Energy (kcal/mol)	Type
MSTRG.6881.4	395	U	UBC	82	Lys	−4.73	DH
	41	C		88	Thr	−2.94	DH
	41	C		90	Thr	−0.75	DH

## Data Availability

The original contributions presented in the study are included in the article, further inquiries can be directed to the corresponding author.
